# Implante Valvar Transcateter em Pacientes com Estenose Valvar Aórtica: Uma *Overview* de Revisões Sistemáticas e Metanálise Incluindo Múltiplas Populações

**DOI:** 10.36660/abc.20220701

**Published:** 2023-06-28

**Authors:** Henrique Diegoli, Marcia Regina Dias Alves, Lucas Miyake Okumura, Caroline Kroll, Dayane Silveira, Luiz Henrique Picolo Furlan

**Affiliations:** 1 Academia VBHC Educação e Consultoria Ltda São Paulo SP Brasil Academia VBHC Educação e Consultoria Ltda , São Paulo , SP – Brasil; 2 Edwards Lifesciences Corporation São Paulo SP Brasil Edwards Lifesciences Corporation , São Paulo , SP – Brasil; 3 Universidade de York York Reino Unido Universidade de York , York – Reino Unido; 4 Universidade da Região de Joinville Joinville SC Brasil Universidade da Região de Joinville , Joinville , SC – Brasil; 5 Unimed do Paraná Curitiba PR Brasil Unimed do Paraná , Curitiba , PR – Brasil

**Keywords:** Substituição da Valva Aórtica Transcateter, Revisão Sistemática, Estenose da Válvula Aórtica

## Abstract

**Fundamento:**

Ensaios clínicos randomizados (ECRs) e estudos observacionais compararam a eficácia e a segurança do implante valvar transcateter (TAVR) e da substituição cirúrgica da valva aórtica (SAVR) em pacientes com estenose aórtica grave.

**Objetivos:**

Comparar TAVR e SAVR em pacientes com diferentes riscos cirúrgicos, características da população e diferentes válvulas protéticas transcateter.

**Métodos:**

Uma *overview* das revisões sistemáticas (RSs) foi realizada seguindo um protocolo estruturado. Os resultados foram agrupados por risco cirúrgico, características da população e diferentes válvulas. Os ECRs foram reanalisados por meio de metanálises nas RSs, e os resultados foram resumidos por meio do método GRADE. O nível de significância estatística adotado foi de 5%.

**Resultados:**

Em comparação com a SAVR, os pacientes com alto risco cirúrgico submetidos à TAVR tiveram um risco menor de ( *odds ratio* , intervalo de confiança de 95%, diferença absoluta de risco) fibrilação atrial (FA) (0,5, 0,29-0,86, -106/1000) e hemorragia com risco à vida (0,29, 0,2-0,42, -215/1000). Pacientes com risco cirúrgico intermediário apresentaram menor risco de FA (0,27, 0,23-0,33, -255/1.000), hemorragia com risco à vida (0,15, 0,12-0,19, -330/1.000) e insuficiência renal aguda (IRA) (0,4, 0,26-0,62, -21/1000). Pacientes com baixo risco cirúrgico tiveram menor risco de morte (0,58, 0,34-0,97, -16/1000), acidente vascular encefálico (AVE) (0,51, 0,28-0,94, -15/1000), FA (0,16, 0,12-0,2, -295/1000), hemorragia com risco à vida (0,17, 0,05-0,55, -76/1000) e IRA (0,27, 0,13-0,55, -21/1000) e tiveram maior risco de implante de marca-passo definitivo (IMD) (4,22, 1,27 -14.02, 141/1000). Os dispositivos de geração mais recente tiveram um risco menor de FA em comparação com as gerações mais antigas, e pacientes usuários de dispositivos expansíveis por balão não apresentaram riscos maiores de IMD.

**Conclusões:**

Este artigo apresenta evidências de que pacientes com risco cirúrgico baixo, intermediário e alto apresentam melhores desfechos quando tratados com TAVR em comparação com a SAVR.

## Introdução e objetivo

Após instalada, a história natural da estenose aórtica grave sintomática evolui para óbito em poucos anos. ^1^ Nenhum tratamento farmacológico é capaz de modificar a história natural da estenose aórtica grave, mas a substituição valvar pode aumentar a sobrevida em cinco anos para mais de 70% dos casos. ^2^

As opções de tratamento para estenose aórtica grave são a substituição cirúrgica da valva aórtica (SAVR) ou o implante valvar transcateter (TAVR), que apresentam diferentes riscos de complicações, incluindo hemorragia, acidente vascular encefálico, necessidade de implante de marca-passo definitivo (IMD) e fibrilação atrial (FA) persistente. ^3 , 4^

Até o momento, ensaios clínicos randomizados (ECRs) e estudos observacionais investigaram a eficácia do TAVR em comparação com a SAVR em pacientes com estenose aórtica grave. No entanto, o corpo de evidências nunca foi resumido em uma visão geral que incluísse grupos populacionais e perfis de risco específicos, além de diferentes válvulas transcateter.

Esta overview das revisões sistemáticas (RSs) resume a RS publicada sobre a eficácia e segurança do TAVR em comparação com a SAVR, facilitando a tomada de decisão ao escolher entre TAVR e SAVR ou entre dispositivos TAVR. Este artigo descreve e compara as evidências que comparam TAVR e SAVR em pacientes com estenose aórtica grave, avaliando a consistência dos achados em diferentes populações, categorias de risco e válvulas protéticas transcateter. Este estudo visa responder qual opção apresenta a melhor taxa de mortalidade e outros desfechos em diferentes grupos de população.

## Métodos

Uma overview de RS foi realizada de acordo com os métodos descritos no Manual Cochrane de Revisões Sistemáticas. ^5^ A revisão sistemática foi concluída seguindo as Diretrizes Brasileiras sobre Revisões Sistemáticas. ^6^ O relato dos achados seguiu os critérios estabelecidos pelo PRISMA. ^7^

A identificação dos artigos e os critérios de exclusão seguiram um protocolo estruturado com três revisores seguindo os mesmos critérios de inclusão e exclusão dos artigos. O presente trabalho inclui estudos envolvendo pacientes com estenose valvar grave que necessitam de um procedimento de substituição valvar, excluindo pacientes inoperáveis, e comparando o TAVR (ou um modelo de válvula específico) com SAVR (ou outro modelo de válvula). Os desfechos em um ano foram mortalidade geral, acidente vascular encefálico, implante de marca-passo definitivo, classificação NYHA ≥2 e os desfechos em 30 dias foram hemorragia com risco à vida e insuficiência renal aguda. Na ausência de dados de um ano, desfechos de 30 dias foram coletados. O estudo consistiu em uma revisão sistemática que incluiu ensaios clínicos randomizados ou estudos observacionais com correspondência de escore de propensão.

Para fins de triagem dos artigos, os registros bibliográficos foram compilados no Mendeley Desktop (versão 1.19.8). Após a exclusão das duplicatas, os títulos e resumos dos artigos foram analisados por dois revisores independentes. Todos os artigos incluídos nesta fase por pelo menos um revisor foram lidos na íntegra por dois revisores. As discrepâncias nesta fase foram resolvidas por meio de discussões entre os revisores. Utilizou-se o Microsoft Excel 365 para registrar a extração dos dados. A extração dos resultados da metanálise foi realizada por um revisor e verificada por um segundo revisor. Quando disponível, as diferenças de subgrupo foram extraídas.

### Descrição dos achados das RSs

Os resultados foram descritos de acordo com o grupo populacional de pacientes. Os resultados foram descritos conforme relatado nas revisões originais, incluindo intervalos de confiança, número de estudos primários, participantes e, quando disponíveis, características da população.

A qualidade metodológica das RSs foi avaliada por dois revisores independentes usando a escala AMSTAR-2. ^8^ Os itens 4 (uso de estratégia de busca abrangente) e 9 (uso de técnica satisfatória para avaliar o risco de viés de estudos individuais) foram considerados críticos.

### Metanálises de ECRs

Independentemente do perfil de risco cirúrgico do paciente, os dados primários de todos os ECRs que comparam os desfechos de TAVR e SAVR foram reanalisados em metanálises por meio do software Review Manager 5.4.1 (Copenhagen: The Nordic Cochrane Centre, The Cochrane Collaboration, 2014). O nível de significância estatística adotado foi de 5%, sendo medido o efeito do *odds ratio* com intervalo de confiança de 95% (IC de 95%) e modelo de efeitos aleatórios, que fornecem intervalos de confiança mais amplos, considerando a incerteza associada à heterogeneidade, o que torna os resultados mais generalizáveis. A metanálise foi realizada pelo método de Mantel-Haenszel. O cálculo de I ^2^ foi utilizado para avaliar a heterogeneidade estatística. A ferramenta RoB-2 foi aplicada a cada ECR para avaliar o risco de viés nos estudos primários. ^9^ O viés de publicação não foi avaliado devido ao pequeno número de estudos incluídos em cada comparação.

Os resultados foram apresentados em tabelas de resumo com uma avaliação da qualidade da evidência usando o método GRADE. ^10^ O método GRADE usa cinco itens: risco de viés, inconsistência, caráter indireto, imprecisão e viés de publicação. O nível de evidência inicia alto para cada desfecho e é reduzido na presença de risco de fraqueza dos achados devido a um dos cinco itens.

## Resultados

**Figura 1:**
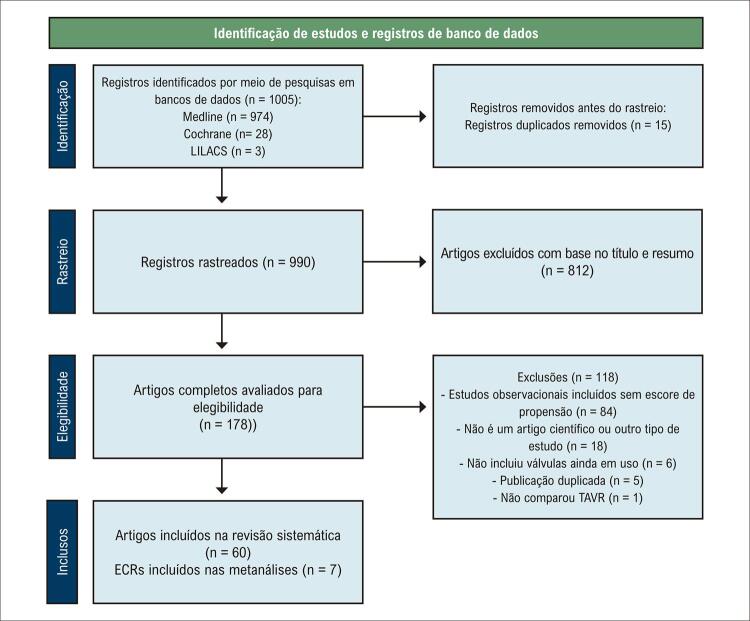
– Fluxograma PRISMA do processo de seleção de estudos. TAVR: implante valvar transcateter; ECRs: ensaios clínicos randomizados.

A pesquisa de literatura resultou em 1.005 registros, dos quais 990 permaneceram após a exclusão das duplicatas ( Figura 1 ). Após a leitura dos títulos e resumos, 812 artigos foram excluídos, resultando em 178 selecionados para leitura completa e, com base nos critérios de seleção, 60 RSs foram incluídas para revisão. A avaliação da qualidade indicou que 19 (31,7%) RSs foram classificadas como de qualidade moderada, 28 (46,7%) de qualidade baixa e 13 (21,7%) de qualidade criticamente baixa ( Tabela Suplementar 1 ).

As RSs incluídas foram 60 artigos científicos que relataram achados de estudos primários, dos quais sete eram ECRs e 40 eram estudos observacionais com correspondência de escore de propensão (PSM, ou *Propensity Score Matching* ). Os dados dos sete ECRs foram incorporados a metanálises para resumir os desfechos por grupo de risco, ^11 - 17^ enquanto os resultados diretos das RSs, que incluíam informações de estudos observacionais e ECRs, foram usados para criar resumos narrativos para os demais grupos populacionais. Uma alta qualidade metodológica foi observada nos ECRs, com especificações disponíveis na Tabela Suplementar 2 .

### Grupos populacionais classificados por risco cirúrgico: metanálise de sete ECRs

#### Pacientes com alto risco cirúrgico

As RSs que comparam pacientes de alto risco foram baseadas nos ECRs PARTNER A (2011) ^11^ e US CoreValve High Risk (2014), ^12^ que usaram as válvulas Sapien (Edwards Lifesciences, Irvine, CA, EUA) e CoreValve (Medtronic, Minneapolis, MN, EUA), respectivamente. Algumas RSs incluíram o estudo STACCATO, mas este estudo não resumiu seus dados, pois foi interrompido prematuramente devido a complicações e à inclusão de apenas 70 pacientes. ^18^ Esses estudos incluíram pacientes com uma chance estimada de morte ou complicações irreversíveis maior que 15% dentro de 30 dias após a cirurgia, tendo como referência o escore da STS. O escore da STS não contempla todas as variáveis que podem ser utilizadas para calcular o risco cirúrgico; portanto, a determinação final de alto risco cirúrgico é feita pelos cirurgiões de cada centro de estudo. O escore STS médio foi de 11,8% (PARTNER A) e 7,3% (US Core Valve High Risk).

A Tabela 1 resume os resultados da metanálise de estudos primários e síntese de evidências usando o método GRADE, que mostram uma redução no risco de FA persistente e hemorragia com risco à vida para pacientes tratados com TAVR. Nenhuma diferença estatisticamente significativa foi observada em outros desfechos ( Figura Central ).

**Tabela 1:** – Síntese dos resultados: pacientes com alto risco cirúrgico

Desfechos	Participantes (estudos)	Qualidade da evidência (GRADE)	Efeito relativo (IC de 95%)	Efeito absoluto
Mortalidade (1 ano)	1381 * †	Moderado ‡	OR de 0,9 (0,7-1,17)	18 a menos a cada 1000 (55 a menos a 28 a mais)
AVE (1 ano)	1262 * †	Baixo ‡ §	OR de 1,06 (0,3-3,7)	5 a menos a cada 1000 (59 a menos a 173 a mais)
FA (1 ano)	1446 * †	Moderado ‡	OR de 0,5 (0,29-0,86)	106 a menos para cada 1000 (27 a 160)
Implante de marca-passo definitivo (1 ano)	1446 * †	Moderado ‡	OR de 1,78 (0,94-3,37)	52 a menos a cada 1000 (34 a menos a 119 a mais)
NYHA >=2 (1 ano)	669 *	Baixo ‡ //	OR de 0,85 (0,62-1,15)	40 a menos a cada 1000 (119 a menos a 34 a mais)
Hemorragia com risco à vida (30 dias)	747 *	Moderado //	OR de 0,29 (0,2-0,42)	215 a menos para cada 1000 (166 a 253)
Insuficiência renal aguda (30 dias)	1446 * †	Muito baixo ‡ // ¶	OR de 0,57 (0,2-1,61)	39 a menos a cada 1000 (75 a menos a 50 a mais)

AVE: acidente vascular cerebral; FA: fibrilação atrial; NYHA: New York Heart Association. * US CoreValve High Risk 2014. † PARTNER 2011. ‡ Inexatidão devido ao grande intervalo de confiança e/ou nenhum efeito do tratamento. § Inconsistência, uma vez que houve redução do risco de acidente vascular encefálico no estudo US CoreValve High Risk, sem diferença estatisticamente significativa no estudo PARTNER A. Heterogeneidade inexplicável entre os estudos identificados (I2 87%, valor de p [p = 0,006]). // Limitações metodológicas, uma vez que não é possível excluir o viés de medição pela impossibilidade de obter pacientes e equipes de caráter cego. A avaliação funcional e a gravidade da hemorragia foram consideradas desfechos não objetivos e, portanto, o nível de evidência foi reduzido. ¶ Inconsistência, uma vez que houve redução do risco de insuficiência renal aguda no estudo US CoreValve High Risk, sem diferença estatisticamente significativa no estudo PARTNER A. Heterogeneidade inexplicável entre os estudos identificados (I2 80%, valor de p [p = 0,02]).

**Figura Central:**
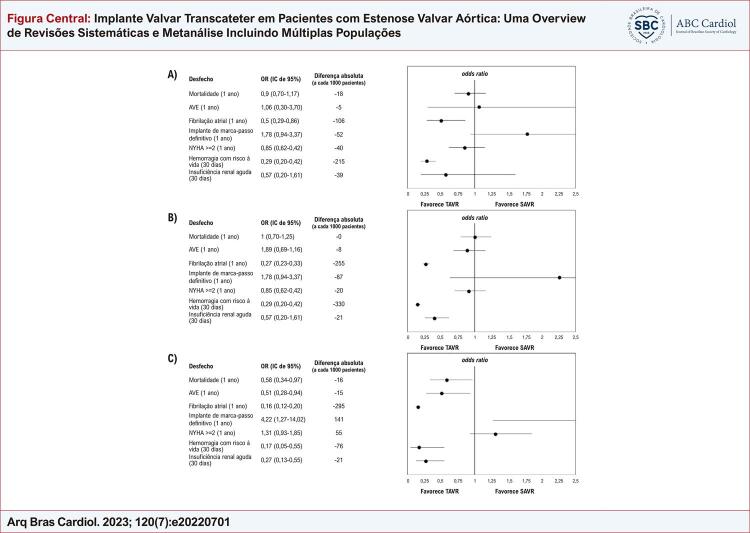
: Implante Valvar Transcateter em Pacientes com Estenose Valvar Aórtica: Uma Overview de Revisões Sistemáticas e Metanálise Incluindo Múltiplas Populações

Resumo das conclusões do estudo entre grupos de risco. A: Alto risco cirúrgico; B: Risco cirúrgico intermediário; C: Baixo risco cirúrgico. NYHA: New York Heart Association.

#### Pacientes com risco cirúrgico intermediário

Os ECRs que incluíram pacientes com risco cirúrgico intermediário foram o PARTNER IIA, ^14^ que utilizou a válvula Sapien XT, e o SURTAVI, ^15^ que utilizou as válvulas CoreValve (84%) e Evolut R (16%). O risco médio de morte em 30 dias dos pacientes incluídos no estudo, calculado pelo escore STS, foi de 5,8% e 4,4%, respectivamente.

Conforme observado na população de alto risco cirúrgico, houve uma redução substancial no risco de FA e hemorragia com risco à vida, conforme mostrado na Tabela 2 . Uma redução no risco de IRA também foi observada. Não houve diferença significativa no risco de IMD. Essa análise foi inconsistente, uma vez que o estudo SURTAVI indicou um aumento substancial no risco (OR de 4,3), e o estudo Partner IIA não indicou uma diferença estatisticamente significativa (I ^2^ = 97%, p < 0,0001).

**Tabela 2:** – Síntese dos resultados: pacientes com risco cirúrgico intermediário

Desfechos	Participantes (estudos)	Qualidade da evidência (GRADE)	Efeito relativo (IC de 95%)	Efeito absoluto
Mortalidade (1 ano)	3222 * †	Alta	OR de 1 (0,79-1,25)	Nenhuma diferença (-22 a +26 a cada 1000)
AVE (1 ano)	3707 * †	Moderado ‡	OR de 0,89 (0,69-1,16)	8 a menos para cada 1000 (-22 a +11)
FA (1 ano)	3692 * †	Alta	OR de 0,27 (0,23-0,33)	255 a menos para cada 1000 (234 a 269)
Implante de marca-passo definitivo (1 ano)	3692 * †	Baixo ‡ §	OR de 2,26 (0,63-8,03)	87 a mais para cada 1000 (-29 a +338)
NYHA >=2 (1 ano)	1120 *	Baixo ‡ //	OR de 0,91 (0,7-1,17)	20 a menos para cada 1000 (-71 a +35)
Hemorragia com risco à vida (30 dias)	2032 †	Moderado //	OR de 0,15 (0,12-0,19)	330 a menos para cada 1000 (306 a 349)
Insuficiência renal aguda (30 dias)	3692 * †	Moderado //	OR de 0,4 (0,26-0,62)	21 a menos a cada 1000 (13 a 27)

AVE: acidente vascular cerebral; FA: fibrilação atrial; NYHA: New York Heart Association. * SURTAVI 2017; † PARTNER 2 2016; ‡ Inexatidão devido ao grande intervalo de confiança e/ou nenhum efeito do tratamento; § Inconsistência, pois não houve risco aumentado de implante de marca-passo definitivo com TAVR no estudo PARTNER 2, mas houve um aumento substancial do risco no estudo SURTAVI. Heterogeneidade significativa identificada (I2 97%, valor p [p < 0,0001]). // Limitações metodológicas, uma vez que não é possível excluir o viés de medição pela impossibilidade de obter pacientes e equipes de caráter cego. A avaliação funcional e a gravidade da hemorragia foram consideradas desfechos não objetivos e, portanto, o nível de evidência foi reduzido.

#### Pacientes com baixo risco cirúrgico

Os ECRs que compararam TAVR e SAVR em pacientes com baixo risco cirúrgico foram o PARTNER III, ^16^ usando a válvula Sapien 3, o Evolut Low Risk, ^17^ usando CoreValve (3,6%), Evolut R (74,1%) e Evolut Pro (22,3%) e o NOTION, ^18^ usando CoreValve. Os escores STS tiveram uma média de 1,9%, 1,9% e 3%, respectivamente.

A Tabela 3 indica os achados da metanálise e a avaliação de qualidade usando o sistema GRADE. O risco de morte, acidente vascular encefálico, FA, hemorragia com risco à vida e IRA diminuiu significativamente no grupo TAVR. Houve aumento do risco de IMD, mas com importante grau de heterogeneidade (I ^2^ 90%, p = 0,02), uma vez que houve aumento significativo do risco no estudo PARTNER III, mas foi observado aumento significativo nos estudos Evolut Low Risk e NOTION.

**Tabela 3:** – Síntese dos resultados: pacientes com baixo risco cirúrgico

Desfechos	Participantes (estudos)	Qualidade da evidência (GRADE)	Efeito relativo (IC de 95%)	Efeito absoluto
Mortalidade (1 ano)	2014 * † ‡	Moderado §	OR de 0,58 (0,34-0,97)	16 a menos para cada 1000 (1 a 26)
AVE (1 ano)	2014 * † ‡	Moderado §	OR de 0,51 (0,28-0,94)	15 a menos para cada 1000 (2 a 22)
FA (1 ano)	2014 * † ‡	Alta	OR de 0,16 (0,12-0,2)	295 a menos para cada 1000 (275 a 316)
Implante de marca-passo definitivo (1 ano)	2014 * † ‡	Baixo § //	OR de 4,22 (1,27-14,02)	141 a mais para cada 1000 (14 a 391)
NYHA >=2 (1 ano)	1909 * † ‡	Baixo § #	OR de 1,31 (0,93-1,85)	55 a mais para cada 1000 (-10 a 107)
Hemorragia com risco à vida (30 dias)	2353 * †	Baixo § #	OR de 0,17 (0,05-0,55)	76 a menos a cada 1000 (40 a 88)
Insuficiência renal aguda (30 dias)	2633 * † ‡	Baixo § #	OR de 0,27 (0,13-0,55)	21 a menos a cada 1000 (13 a 25)

AVE: acidente vascular cerebral; FA: fibrilação atrial; NYHA: New York Heart Association. * PARTNER 3. † Evolut Low Risk. ‡ NOTION. § Imprecisão devido ao grande intervalo de confiança e/ou nenhum efeito do tratamento. // Inconsistência, pois o PARTNER 3 não apresentou risco aumentado de implante de marca-passo definitivo com TAVR, mas o Evolut Low Risk e o Notion, sim. Heterogeneidade inexplicável entre os estudos (I2 90%, valor de p [p = 0,02]). ¶ Inconsistência devido à amplitude do intervalo de confiança. # Limitações metodológicas: como é impossível obter pacientes e equipes de caráter cego, o viés de medição não pode ser eliminado. A avaliação funcional e a gravidade da hemorragia foram consideradas desfechos não objetivos e, portanto, o nível de evidência foi reduzido.

## Outros grupos populacionais: visão geral das revisões sistemáticas

### Diferenças entre sexos

Duas metanálises compararam as diferenças de sexo nos desfechos de TAVR e SAVR, com níveis de confiança moderados na escala AMSTAR-2 ( Tabela Suplementar 3 ). ^19 , 20^ Os estudos identificaram que o TAVR em mulheres estava associado a uma redução no risco de morte e IRA, mas não em homens. No entanto, o TAVR aumentou significativamente o risco de IMD em homens, mas não em mulheres. ^20^

### Cirurgia cardíaca prévia

Latif et al., 2021 (nível de confiança moderado), ^21^ compararam TAVR e SAVR em pacientes com cirurgia cardíaca prévia ( tabela Suplementar 3 ). Os resultados não mostraram diferenças significativas no risco de morte ou IRA, mas o TAVR reduziu significativamente o risco de acidente vascular encefálico e hemorragia grave.

### Diferentes válvulas protéticas transcateter

Zhang et al., 2020 (nível de confiança moderado), ^22^ comparam os desfechos de TAVR e SAVR em diversos subgrupos, incluindo gerações mais antigas em comparação com gerações novas e dispositivos de expansão de balão em comparação com dispositivos autoexpansíveis, incluindo sete ECRs (7.771 pacientes). Em comparação com as gerações mais antigas, as gerações mais novas não reduziram o risco de morte, acidente vascular encefálico ou IRA, mas reduziram o risco de FA e IMD ( tabela Suplementar 3 ). ^22^ Além disso, Zhang et al., 2020 , ^22^ compararam os desfechos entre sistemas expansíveis por balão e aqueles autoexpansíveis. Os dispositivos expansíveis por balão demonstraram riscos significativamente reduzidos de IMD e hemorragia grave em 30 dias, enquanto os dispositivos autoexpansíveis apresentaram menor incidência de IRA.

Gozdek et al., 2020 (baixo nível de confiança), ^23^ comparou as diferenças de desfechos entre o sistema autoexpansível ACURATE neo (Boston Scientific Corporation, Marlborough, MA, EUA) e o sistema expansível por balão SAPIEN 3, em um ECR e cinco estudos observacionais com PSM, incluindo 2.818 participantes. O risco de morte em 30 dias foi maior em pacientes usando o ACURATE neo em comparação com o SAPIEN 3 ( Tabela Suplementar 3 ), consistente com a falha do ECR SCOPE I em comprovar a não inferioridade do ACURATE neo em relação ao SAPIEN 3. Embora o ECR SCOPE I não tenha observado diferença significativa na necessidade de IMD, a análise de estudos observacionais com PSM indicou menor necessidade no grupo que utilizou o sistema ACURATE neo.

Alperi et al., 2020 (baixo nível de confiança), ^24^ realizaram uma metanálise que incluiu 35 estudos observacionais com PSM ou ECRs buscando investigar o papel de múltiplos fatores na ocorrência de IMD. Na comparação entre Sapien 3 e Evolut R/PRO, envolvendo 23.965 pacientes em quatro estudos observacionais com PSM e um ECR, observou-se maior frequência de IMD com Evolut R/PRO nos estudos observacionais com PSM, com diferença estatisticamente significativa em três dos quatro estudos. No entanto, não houve diferença significativa no ECR. Na comparação entre Sapien 3 e ACURATE neo, 2.194 pacientes foram estudados em quatro estudos observacionais com PSM e um ECR. Nos estudos observacionais com PSM, houve maior frequência de IMD no grupo que utilizou o Sapien 3, entre os quais dois apresentaram diferença estatisticamente significativa, enquanto o ECR (SCOPE I) não observou diferença significativa. Apenas um estudo observacional com PSM, com 251 pacientes, comparou o IMD no sistema Evolut Pro vs. ACURATE neo, não observando diferenças significativas. Os sistemas Portico e Sapien 3 foram comparados em um estudo observacional com PSM com 177 pacientes, que também não encontrou diferenças significativas. O sistema Portico também foi comparado a um grupo de válvulas disponíveis comercialmente em um ECR de 732 pacientes, predominantemente expansível por balão, com um desfecho desfavorável para o Portico.

## Discussão

O uso de TAVR foi associado a uma redução do risco de FA persistente e hemorragia com risco à vida em pacientes em qualquer nível de risco cirúrgico. No entanto, a redução da mortalidade foi observada apenas em pacientes de baixo risco cirúrgico, não naqueles de risco cirúrgico intermediário ou alto. Além disso, pacientes submetidos a TAVR com risco cirúrgico baixo ou intermediário apresentaram menor risco de IRA. Por outro lado, o TAVR foi associado a um risco maior de IMD, mas a diferença foi estatisticamente significativa apenas nos casos de válvulas protéticas autoexpansíveis transcateter.

A diminuição na probabilidade de FA e IMD com válvulas de segunda geração provavelmente se deve a múltiplos fatores, incluindo o envolvimento de pacientes de menor risco em estudos dessas válvulas, maior proficiência das equipes cirúrgicas, aprimoramentos nas técnicas e atributos distintos de válvulas de segunda geração que permitem um implante mais preciso.

Embora tenha havido maior incidência de IMD no grupo TAVR, ela foi altamente heterogênea porque a família de estudos PARTNER com a válvula expansível por balão Sapien não apresentou aumento no risco de IMD. Em estudos comparando diferentes válvulas protéticas transcateter, as válvulas autoexpansíveis foram associadas a um risco substancialmente maior de IMD. Em comparação com o Sapien 3, as válvulas Evolut R e Portico apresentaram maior risco de IMD. A exceção foi a válvula ACURATE neo, uma válvula autoexpansível com menor risco de IMD, mas maior risco de morte.

A SAVR é uma opção terapêutica frequentemente utilizada em pacientes com estenose valvar aórtica grave, devido à sua história natural de prognóstico ruim, com óbito ocorrendo em poucos anos. ^1^ Os primeiros estudos para investigar a eficácia e segurança do TAVR foram realizados em pacientes inoperáveis, mostrando uma redução significativa da mortalidade quando comparada ao tratamento médico, ^25^ assim como em pacientes de alto risco cirúrgico, nos quais o TAVR apresentou um risco de morte semelhante à SAVR. ^26 , 27^ Como observado neste estudo, os ECRs mais recentes apresentam fortes evidências da segurança e eficácia do procedimento. Três fatores podem explicar os melhores desfechos em perfis de risco mais baixos: diferentes gerações de válvulas foram usadas em múltiplos estudos pivotais; acesso menos invasivo utilizado em estudos mais recentes (transfemoral em vez de transapical); melhorias da experiência das equipes, imagens e outros fatores nos últimos anos.

As evidências sugerem um benefício absoluto substancial em pacientes de risco cirúrgico intermediário e baixo, ainda maior do que aquele identificado em pacientes de alto risco cirúrgico. Sendo assim, os achados são de grande importância para informar uma atualização sobre as recomendações dos órgãos de saúde sobre a ampliação do acesso ao procedimento no Brasil e em outros países.

Atualmente, o TAVR é recomendado apenas para pacientes inoperáveis ou com alto risco cirúrgico pela Agência Nacional de Saúde Suplementar (ANS). ^28^ No sistema público de saúde do Brasil, o TAVR é recomendado para pacientes inoperáveis. ^29^

Em contraste, outras diretrizes recomendam o uso mais amplo do TAVR, como aquelas da American Heart Association e do American College of Cardiology (tratamento de preferência para pacientes acima de 80 anos), ^3^ Diretrizes da European Society of Cardiology (ESC) e a European Association of Cardio-Thoracic Surgery (EACTS) (TAVR recomendado para pacientes com idade superior a 75 anos ou com alto risco cirúrgico). ^30^ As Diretrizes para Valvopatia da Sociedade Brasileira de Cardiologia sugerem o TAVR como a opção preferencial para pacientes acima de 70 anos, independentemente do risco cirúrgico, mas a SAVR pode ser preferível para perfis de risco baixo e intermediário, de acordo com uma decisão da equipe. ^4^

Futuras comparações entre TAVR e SAVR também devem considerar a redução significativa do risco de FA, condição que, na maioria das vezes, requer anticoagulação de longo prazo associada a altos custos com medicamentos, exames de sangue e consultas médicas. A redução da incidência de acidentes vasculares encefálicos também impacta positivamente os custos com internações hospitalares e o acompanhamento a longo prazo. Da mesma forma, reduzir a incidência de hemorragia com risco à vida diminui os custos hospitalares relacionados ao procedimento.

### Pontos fortes e limitações

Esta é a primeira overview de revisões sistemáticas que investiga o TAVR em comparação com a SAVR, incluindo grupos específicos de população e diferentes válvulas protéticas transcateter de TAVR, totalizando 60 RSs na análise final. Essa abordagem permite uma visão abrangente das evidências científicas disponíveis sobre o procedimento, oferecendo um resumo das informações necessárias aos profissionais de saúde e gestores de saúde pública para a tomada de decisões.

O desenho do estudo é limitado pelo uso de informações de fontes secundárias (outras revisões sistemáticas), que foram reduzidas pelo resumo de evidências por meio do sistema GRADE usando artigos primários.

## Conclusões

Uma overview de 60 RSs e metanálise de sete ECRs identificou que o TAVR foi associado a desfechos significativamente melhores em comparação com a SAVR, exceto pelo risco de IMD, com evidência de qualidade moderada a baixa. Um aumento substancial do risco de IMD foi observado nas próteses autoexpansíveis, o que não ocorreu nas válvulas expansíveis por balão. Estudos mais recentes, que incluíram pacientes com riscos baixo e intermediário, demonstraram um maior benefício absoluto do TAVR, em comparação com a SAVR, do que os estudos que incluíram pacientes com risco mais alto. Esses achados ajudam a informar as autoridades sobre a expansão da indicação do TAVR, embasadas em evidências científicas sólidas e recomendações da sociedade médica.

## * Material suplementar

Para informação adicional, por favor, <ext-link ext-link-type="uri" xlink:href="http://abccardiol.org/supplementary-material/2023/12007/2022-0701-Supplementary-material.pdf">clique aqui</ext-link>.
