# Supplementary material for: Implante Valvar Transcateter em Pacientes com Estenose Valvar Aórtica: Uma Overview de Revisões Sistemáticas e Metanálise Incluindo Múltiplas Populações
Source: Arq Bras Cardiol. 2023 Jun 28;120(7):e20220701. [Article in Portuguese] doi: 10.36660/abc.20220701 (PMC10365004; doi:10.36660/abc.20220701)
Supplement: Translation

# Translation: Transcatheter Valve Replacement in Patients with Aortic Valve Stenosis: An Overview of Systematic Reviews and Meta-Analysis with Different Populations

**Authors:** Henrique Diegoli, Marcia Regina Dias Alves, Lucas Miyake Okumura, Caroline Kroll, Dayane Silveira, Luiz Henrique Picolo Furlan

**Affiliations:** 1 Brazil Academia VBHC Educação e Consultoria Ltda, São Paulo, SP – Brazil; 2 Brazil Edwards Lifesciences Corporation, São Paulo, SP – Brazil; 3 United Kingdom Universidade de York, York – United Kingdom; 4 Brazil Universidade da Região de Joinville, Joinville, SC – Brazil; 5 Brazil Unimed do Paraná, Curitiba, PR – Brazil

**Keywords:** Transcatheter Aortic Valve Replacement, Systematic Review, Aortic Valve Stenosis

## Abstract

**Background:**

Randomized controlled trials (RCTs) and observational studies have compared the efficacy and safety of transcatheter aortic valve replacement (TAVR) and surgical aortic valve replacement (SAVR) in patients with severe aortic stenosis.

**Objectives:**

Compare TAVR and SAVR in patients with different surgical risks, population characteristics, and different transcatheter prosthetic valves.

**Methods:**

An overview of systematic reviews (SRs) was conducted following a structured protocol. Results were grouped by surgical risk, population characteristics, and different valves. RCTs in the SRs were reanalyzed through meta-analyses, and the results were summarized using the GRADE method. The adopted level of statistical significance was 5%.

**Results:**

Compared to SAVR, patients with high surgical risk using TAVR had a lower risk of (odds ratio, 95% confidence interval, absolute risk difference) atrial fibrillation (AF) (0.5, 0.29-0.86, -106/1000) and life-threatening bleeding (0.29, 0.2-0.42, -215/1000). Patients with intermediate surgical risk had a lower risk of AF (0.27, 0.23-0.33, -255/1000), life-threatening bleeding (0.15, 0.12-0.19, -330/1000), and acute renal failure (ARF) (0.4, 0.26-0.62, -21/1000). Patients with low surgical risk had a lower risk of death (0.58, 0.34-0.97, -16/1000), stroke (0.51, 0.28-0.94, -15/1000), AF (0.16, 0.12-0.2, -295/1000), life-threatening bleeding (0.17, 0.05-0.55, -76/1000), and ARF (0.27, 0.13-0.55, -21/1000), and had a higher risk of permanent pacemaker implantation (PPI) (4.22, 1.27-14.02, 141/1000). Newer generation devices had a lower risk of AF than older generations, and patients using balloon-expandable devices did not experience higher risks of PPI.

**Conclusions:**

This paper provides evidence that patients at low, intermediate, and high surgical risks have better outcomes when treated with TAVR compared with SAVR.

## Introduction and objective

Once installed, the natural history of symptomatic severe aortic stenosis is to evolve to death within a few years.^1^ No drugs can modify the natural history of severe aortic stenosis, but valve replacement can increase five-year survival to above 70%.^2^

The treatment options for severe aortic stenosis are surgical aortic valve replacement (SAVR) or transcatheter aortic valve replacement (TAVR), which have different risks of complications such as hemorrhage, stroke, need for permanent pacemaker implantation (PPI), and persistent atrial fibrillation (AF).^3 , 4^

To date, randomized clinical trials (RCTs) and observational studies have investigated the effectiveness of TAVR compared with SAVR for patients with severe aortic stenosis. However, the body of evidence has never been summarized in an overview that included specific population groups, risk profiles, and different transcatheter valves.

This overview of systematic reviews summarizes the published SR on the effectiveness and safety of TAVR compared to SAVR, facilitating decision-making when choosing between TAVR and SAVR or between TAVR devices. This paper describes and compares the evidence comparing TAVR and SAVR in patients with severe aortic stenosis, evaluating the consistency of findings in different populations, risk categories, and transcatheter prosthetic valves. This study aims to answer which option has the best mortality rate and other outcomes in different population groups.

## Methods

An overview of SR was performed according to the methods described in the Cochrane Handbook of Systematic Reviews.^5^ The systematic review was completed following the Brazilian Guidelines on Systematic Reviews.^6^ The report of the findings followed the criteria set out by PRISMA.^7^

The identification of papers and exclusion criteria followed a structured protocol with three reviewers following the same criteria for the inclusion and exclusion of papers. The present paper includes studies involving patients with severe valve stenosis needing a valve replacement procedure, excludes inoperable patients, and compares TAVR (or a specific valve model) with SAVR (or another valve model). One-year outcomes were overall mortality, stroke, permanent pacemaker implantation, NYHA classification ≥2, and 30-day outcomes were life-threatening bleeding and acute renal failure. In the absence of one-year data, 30-day outcomes were extracted. The type of study was a systematic review that included either randomized controlled trials or observational studies with propensity score matching.

For the purpose of screening papers, bibliographic records were compiled in Mendeley Desktop (version 1.19.8). After excluding duplicates, the titles and abstracts of the papers were screened by two independent reviewers. All papers included in this phase by at least one reviewer were read in full by two reviewers. Discrepancies at this stage were settled through discussions between reviewers. Microsoft Excel 365 was used for registering the data extraction. The extraction of meta-analysis results was performed by one reviewer and verified by a second reviewer. When available, subgroup differences were extracted.

### Description of findings from the SRs

The results were described according to the patient population group. Results were described as reported in the original reviews, including confidence intervals, the number of primary studies, participants, and, where available, population characteristics.

The methodological quality of SRs was assessed by two independent reviewers using the AMSTAR-2 scale.^8^ Items 4 (use of comprehensive search strategy) and 9 (use of satisfactory technique for assessing the risk of bias of individual studies) were considered critical.

### Meta-analyses of RCTs

Regardless of the patient’s surgical risk profile, primary data of all RCTs comparing the outcomes of TAVR and SAVR were reanalyzed in meta-analyses using the Review Manager 5.4.1 software (Copenhagen: The Nordic Cochrane Centre, The Cochrane Collaboration, 2014). The level of statistical significance adopted was 5%, and the odds ratio effect with a 95% confidence interval (95% CI) and random effects model were measured, which provide wider confidence intervals, taking into account the uncertainty associated with heterogeneity, which makes the results more generalizable. The meta-analysis was performed using the Mantel-Haenszel method. The I^2^calculation was used to assess statistical heterogeneity. The RoB-2 tool was applied to each RCT to assess the risk of bias in the primary studies.^9^ Publication bias was not assessed due to the small number of studies included in each comparison.

The results were presented in summary tables with an assessment of the evidence quality using the GRADE method.^10^ The GRADE method uses five items: risk of bias, inconsistency, indirectness, imprecision, and publication bias. The level of evidence starts as high for each outcome and is reduced in the presence of risk of weakness in the findings due to one of the five items.

## Results

The literature search yielded 1,005 records, 990 of which remained after duplicates were deleted ( Figure 1 ). After reading titles and abstracts, 812 papers were excluded, leaving 178 for complete text reading, after which 60 SRs remained for review based on the selection criteria. The quality assessment indicated that 19 (31.7%) SRs were classified as moderate quality, 28 (46.7%) as low quality, and 13 (21.7%) as critically-low quality ( Supplementary Table 1 ).

**Figure 1:** – PRISMA flowchart of the study selection process. TAVR: transcatheter aortic valve replacement; RCTs: Randomized controlled trials.

The SRs were 60 scientific papers reporting findings from primary studies, of which seven were RCTs, and 40 were observational studies with propensity score matching (PSM). The data from the seven RCTs were incorporated into meta-analyses to summarize outcomes by risk group,^11 - 17^ while the direct results of the SRs, which included information from observational studies and RCTs, were used to create narrative summaries for the remaining population groups. High methodological quality was observed in the RCTs, with specifications available in Supplementary Table 2 .

### Population groups classified by surgical risk: meta-analysis of seven RCTs

#### Patients at high surgical risk

The SRs comparing patients at high risk were based on the RCTs PARTNER A (2011)^11^ and US CoreValve High Risk (2014),^12^ which used the Sapien (Edwards Lifesciences, Irvine, CA, USA) and CoreValve valves (Medtronic, Minneapolis, MN, USA), respectively. Some SRs included the STACCATO study, but this study did not summarize its data as it was stopped prematurely due to complications and the inclusion of only 70 patients.^13^ These studies included patients with an estimated chance of death or irreversible complications greater than 15% within 30 days after surgery, using the STS Score as a reference. The STS Score does not include all the variables that can be used to calculate the surgical risk; therefore, the final determination of high operative risk is made by surgeons at each study center. The average STS Score was 11.8% (PARTNER A) and 7.3% (US Core Valve High Risk).

Table 1 summarizes the meta-analysis results from primary studies and evidence synthesis using the GRADE method, showing a reduction in the risk of persistent AF and life-threatening bleeding for patients treated with TAVR. No statistically significant difference was observed in other outcomes ( Central Illustration ).

**Table 1:** – Summary of the results: patients at high surgical risk

Outcomes	Participants (studies)	Quality of the evidence (GRADE)	Relative effect (95% CI)	Absolute effect
Mortality (1 year)	1381 * †	Moderate ‡	OR 0.9 (0.7-1.17)	18 less per 1000 (55 less to 28 more)
Stroke (1 year)	1262 * †	Low ‡ §	OR 1.06 (0.3-3.7)	5 less per 1000 (59 less to 173 more)
AF (1 year)	1446 * †	Moderate ‡	OR 0.5 (0.29-0.86)	106 less per 1000 (27 to 160)
Permanent pacemaker implant (1 year)	1446 * †	Moderate ‡	OR 1.78 (0.94-3.37)	52 less per 1000 (34 less to 119 more)
NYHA >=2 (1 year)	669 *	Low‡ //	OR 0.85 (0.62-1.15)	40 less per 1000 (119 less to 34 more)
Life-threatening bleeding (30 days)	747 *	Moderate //	OR 0.29 (0.2-0.42)	215 less per 1000 (166 to 253)
Acute kidney failure (30 days)	1446 * †	Very low ‡ // ¶	OR 0.57 (0.2-1.61)	39 less per 1000 (75 less to 50 more)

AF: atrial fibrillation; NYHA: New York Heart Association. * US CoreValve High Risk 2014. † PARTNER 2011. ‡ Inaccuracy due to large confidence interval and/or no treatment effect. § Inconsistency, as there was a reduction in the risk of stroke in the US CoreValve High Risk study, with no statistically significant difference in the PARTNER A study. Unexplained heterogeneity between studies identified (I2 87%, p-value [p = 0.006]). // Methodological limitations, as it is not possible to exclude the measurement bias due to the impossibility of obtaining blinding patients and teams. Given that the functional assessment and bleeding severity were considered non-objective outcomes, the level of evidence was reduced. ¶ Inconsistency, as there was a reduction in the risk of acute renal failure in the US CoreValve High Risk study, with no statistically significant difference in the PARTNER A study. Unexplained heterogeneity between studies identified (I2 80%, p-value [p = 0.02]).

**Central Illustration:** : Transcatheter Valve Replacement in Patients with Aortic Valve Stenosis: An Overview of Systematic Reviews and Meta-Analysis with Different Populations

Summary of the findings of the study across risk groups. A: High surgical risk; B: Intermediate surgical risk; C: Low surgical risk. NYHA: New York Heart Association.

#### Patients at intermediate surgical risk

The RCTs that included patients with intermediate surgical risk were PARTNER IIA,^14^ which used the Sapien XT valve, and SURTAVI,^15^ which used both the CoreValve (84%) and Evolut R (16%) valves. The average risk of death within 30 days of the patients included in the study, calculated by the STS Score, was 5.8% and 4.4%, respectively.

As observed in the high surgical-risk population, there was a substantial reduction in the risk of AF and life-threatening bleeding, as shown in Table 2 . A reduction in the risk of ARF was also observed. There was no significant difference in the risk of PP. Such an analysis was inconsistent, as the SURTAVI study indicated a substantial increase in risk (OR 4.3), and the Partner IIA study did not indicate a statistically significant difference (I^2^= 97%, p < 0.0001).

**Table 2:** – Summary of the results: patients at intermediate surgical risk

Outcomes	Participants (studies)	Quality of the evidence (GRADE)	Relative effect (95% CI)	Absolute effect
Mortality (1 year)	3222 * †	High	OR 1 (0.79-1.25)	No difference (-22 to +26 per 1000)
Stroke (1 year)	3707 * †	Moderate ‡	OR 0.89 (0.69-1.16)	8 less per 1000 (-22 to +11)
AF (1 year)	3692 * †	High	OR 0.27 (0.23-0.33)	255 less per 1000 (234 to 269)
Permanent pacemaker implant (1 year)	3692 * †	Low ‡ §	OR 2.26 (0.63-8.03)	87 more per 1000 (-29 to +338)
NYHA >=2 (1 year)	1120 *	Low ‡ //	OR 0.91 (0.7-1.17)	20 less per 1000 (-71 to +35)
Life-threatening bleeding (30 days)	2032 †	Moderate //	OR 0.15 (0.12-0.19)	330 less per 1000 (306 to 349)
Acute kidney failure (30 days)	3692 * †	Moderate //	OR 0.4 (0.26-0.62)	21 less per 1000 (13 to 27)

FA: fibrilação atrial; NYHA: New York Heart Association. * SURTAVI 2017; † PARTNER 2 2016; ‡ Inexatidão devido ao grande intervalo de confiança e/ou nenhum efeito do tratamento; § Inconsistência, pois não houve risco aumentado de implante de marca-passo definitivo com TAVR no estudo PARTNER 2, mas houve um aumento substancial do risco no estudo SURTAVI. Heterogeneidade significativa identificada (I2 97%, valor p [p < 0,0001]). // Limitações metodológicas, uma vez que não é possível excluir o viés de medição pela impossibilidade de obter pacientes e equipes de caráter cego. A avaliação funcional e a gravidade da hemorragia foram consideradas desfechos não objetivos e, portanto, o nível de evidência foi reduzido.

#### Patients at low surgical risk

The RCTs comparing TAVR and SAVR in patients with low surgical risk were PARTNER III,^16^ using Sapien 3, Evolut Low Risk,^17^ using CoreValve (3.6%), Evolut R (74.1%), and Evolut Pro (22.3%), and NOTION,^18^ using CoreValve. The STS Scores averaged 1.9%, 1.9%, and 3%, respectively.

Table 3 indicates the findings of the meta-analysis and quality assessment using the GRADE system. The risk of death, stroke, AF, life-threatening bleeding, and ARF significantly decreased in the TAVR group. There was an increase in the risk of PPI, but with an important degree of heterogeneity (I^2^90%, p = 0.02) since there was no significant risk increase in the PARTNER III study, but a significant increase was observed in the Evolut Low Risk and NOTION studies.

**Table 3:** – Summary of the results: patients at low surgical risk

Outcomes	Participants (studies)	Quality of the evidence (GRADE)	Relative effect (95% CI)	Absolute effect
Mortality (1 year)	2014 * † ‡	Moderate §	OR 0.58 (0.34-0.97)	16 less per 1000 (1 to 26)
Stroke (1 year)	2014 * † ‡	Moderate §	OR 0.51 (0.28-0.94)	15 less per 1000 (2 to 22)
AF (1 year)	2014 * † ‡	High	OR 0.16 (0.12-0.2)	295 less per 1000 (275 to 316)
Permanent pacemaker implant (1 year)	2014 * † ‡	Low § //	OR 4.22 (1.27-14.02)	141 more per 1000 (14 to 391)
NYHA >=2 (1 year)	1909 * † ‡	Low § #	OR 1.31 (0.93-1.85)	55 more per 1000 (-10 to 107)
Life-threatening bleeding (30 days)	2353 * †	Low § #	OR 0.17 (0.05-0.55)	76 less per 1000 (40 to 88)
Acute kidney failure (30 days)	2633 * † ‡	Low § #	OR 0.27 (0.13-0.55)	21 less per 1000 (13 to 25)

AF: atrial fibrillation; NYHA: New York Heart Association. * PARTNER 3. † Evolut Low Risk. ‡ NOTION. § Imprecision due to large confidence interval and/or no treatment effect. // Inconsistency, as PARTNER 3 showed no increased risk of permanent pacemaker implantation with TAVR, but Evolut Low Risk and Notion did. Unexplained heterogeneity between studies (I2 90%, p-value [p = 0.02]). ¶ Inconsistency due to the amplitude of the confidence interval. # Methodological limitations: since blinding patients and teams is impossible, measurement bias cannot be eliminated. Given that the functional assessment and bleeding severity were considered non-objective outcomes, the level of evidence was reduced.

## Other population groups: overview of systematic reviews

### Gender differences

Two meta-analyses compared gender differences in TAVR and SAVR outcomes, with moderate confidence levels in the AMSTAR-2 scale ( Supplementary Table 3 ).^19 , 20^ The studies identified that TAVR in women was associated with a reduction in the risk of death and ARF, but not in men. However, TAVR significantly increased the risk of PPI in men but not in women.^20^

### Previous cardiac surgery

Latif et al., 2021 (moderate confidence level),^21^ compared TAVR and SAVR in patients with previous cardiac surgery ( Supplementary Table 3 ). Results showed no significant differences in the risk of death or ARF, but TAVR significantly reduced the risk of stroke and major bleeding.

### Different transcatheter prosthetic valves

Zhang et al., 2020 (moderate confidence level),^22^ compared the outcomes of TAVR and SAVR in multiple subgroups, including older vs. newer generations and balloon-expanding vs. self-expanding devices, including seven RCTs (7,771 patients). Compared to older generations, newer generations did not reduce the risk of death, stroke, or ARF but reduced the risk of AF and PPI ( Supplementary Table 3 ).^22^Zhang et al., 2020 ,^22^ also compared outcomes between balloon-expandable and self-expanding systems. Balloon-expandable devices showed significantly reduced risks of PPI and 30-day major bleeding, while self-expanding devices had lower incidences of ARF.

Gozdek et al., 2020 (low confidence level),^23^ compared outcomes differences between the ACURATE neo self-expanding system (Boston Scientific Corporation, Marlborough, MA, USA) and the SAPIEN 3 balloon-expandable system, in one RCT and five observational studies with PSM, including 2,818 participants. The 30-day death risk was higher in patients using ACURATE neo compared to SAPIEN 3 ( Supplementary Table 3 ), consistent with the failure of RCT SCOPE I in proving the non-inferiority of ACURATE neo in relation to SAPIEN 3. Although the RCT SCOPE I did not observe a significant difference in the need for PPI, the analysis of observational studies with PSM indicated less need in the group that used ACURATE neo.

Alperi et al., 2020 (low confidence level),^24^ performed a meta-analysis that included 35 observational studies with PSM or RCTs seeking to investigate the role of various factors in the occurrence of PPI. In the comparison between Sapien 3 and Evolut R/PRO, involving 23,965 patients in four observational studies with PSM and one RCT, a higher frequency of PPI was observed with Evolut R/PRO in the observational studies with PSM, with a statistically significant difference in three of the four studies. However, there was no significant difference in the RCT. In the comparison between Sapien 3 and ACURATE neo, 2,194 patients were studied in four observational studies with PSM and one RCT. In the observational studies with PSM, there was a higher frequency of PPI in the group that used Sapien 3, among which two showed a statistically significant difference, while the RCT (SCOPE I) did not observe a significant difference. Only one observational study with PSM with 251 patients compared PPI in Evolut Pro vs. ACURATE neo, not observing significant differences. Portico and Sapien 3 were compared in an observational study with PSM with 177 patients, which also found no significant differences. Portico was also compared to a group of commercially available valves in a 732-patient, predominantly balloon-expandable RCT, with an unfavorable outcome for Portico.

## Discussion

TAVR was found to lower the risk of persistent AF and life-threatening bleeding in patients at any level of surgical risk. However, the reduction in mortality was observed only in patients at low surgical risk, not in those at intermediate or high surgical risk. Moreover, patients undergoing TAVR with low or intermediate surgical risk had a reduced risk of ARF. On the other hand, TAVR was associated with an increased risk of PPI, but statistically significant increases were observed only in cases involving self-expanding transcatheter prostetic valves.

The decrease in the likelihood of AF and PPI with second-generation valves is likely due to multiple factors, such as the involvement of lower-risk patients in studies of these valves, increased proficiency of surgical teams, enhancements in techniques, and distinct attributes of second-generation valves that enable more precise valve implantation.

Although there was a higher incidence of PPI in the TAVR group, it was highly heterogeneous because the family of PARTNER studies using the Sapien balloon-expandable valve did not have an increase in the risk of PPI. In studies comparing different transcatheter prosthetic valves, self-expanding valves were associated with a substantially higher risk of PPI. Compared to Sapien 3, the Evolut R and Portico valves had a higher risk of PPI. The exception was the ACURATE neo valve, a self-expanding valve with a lower risk of PPI but a higher risk of death.

SAVR is a therapeutic option often used in patients with severe aortic valve stenosis, as its natural history has a poor prognosis, with death occurring within a few years.^1^ The first studies to investigate the efficacy and safety of TAVR were conducted in inoperable patients, showing a significant mortality reduction when compared to medical treatment,^25^ as well as in patients at high surgical risk, in whom TAVR presented a risk of death similar to SAVR.^26 , 27^ As observed in this study, the most recent RCTs provide strong evidence of the safety and efficacy of the procedure. Three factors may explain the improved outcomes in lower risks: different valve generations were used in the various pivotal trials; less invasive access used in more recent studies (transfemoral instead of transapical); improvement in the experience of teams, imaging, and other factors in recent years.

The evidence suggests a substantial absolute benefit in patients at intermediate and low surgical risk, even greater than that identified in patients at high surgical risk. Therefore, the findings are of great importance to inform an update of the health agency recommendations about the expansion of access to the procedure in Brazil and other countries.

Currently, TAVR is only recommended for inoperable patients or patients at high surgical risk by Brazil’s National Supplementary Health Agency (ANS).^28^ In Brazil’s public healthcare system, TAVR is recommended for inoperable patients.^29^

In contrast, other guidelines recommend broader use of TAVR, such as the American Heart Association and the American College of Cardiology (treatment of choice for patients over the age of 80),^3^ Guidelines of the European Society of Cardiology (ESC), and the European Association of Cardio-Thoracic Surgery (EACTS) (TAVR is recommended for patients over the age of 75 years or those at high surgical risk).^30^ The Guidelines for Valvular Heart Disease of the Brazilian Society of Cardiology suggests TAVR as the preferred option for patients over the age of 70 regardless of surgical risk, but SAVR may be preferable for low and intermediate risk, according to a Heart Team decision.^4^

Future evaluations comparing TAVR and SAVR should also consider the significant reduction in the risk of AF, a condition that, in most cases, requires long-term anticoagulation associated with high costs of medications, blood tests, and visits to the doctor’s office. The reduction in the incidence of stroke also positively impacts the costs of hospital admissions and long-term follow-up. Likewise, reducing the incidence of life-threatening bleeding decreases the hospital costs related to the procedure.

### Strengths and limitations

This is the first overview of systematic reviews investigating TAVR compared to SAVR, including specific population groups and different transcatheter prosthetic valves of TAVR, totaling 60 SRs in the final analysis. This approach allows for a comprehensive view of the scientific evidence available about the procedure, providing a summary of information needed by health professionals and public health managers to make decisions.

The study design is limited by using information from secondary sources (other systematic reviews), which was reduced by the summary of evidence through the GRADE system using primary papers.

## Conclusions

An overview of 60 SRs and meta-analysis of seven RCTs identified that TAVR was associated with significantly better outcomes than SAVR, except for the risk of PPI, with moderate to low-quality evidence. A substantial increase in the risk of PPI was identified in self-expanding prostheses, which did not occur in the balloon-expandable valves. More recent trials, which included patients at low and intermediate risks, demonstrate a greater absolute benefit from TAVR, compared with SAVR, than trials that included patients at a higher risk. These findings help inform authorities about the expansion of TAVI’s indication, supported by solid scientific evidence and medical society recommendations.

## *Supplemental Materials

For additional information, please click here .
